# Assessing the feasibility of a web-based registry for multiple orphan lung diseases: the Australasian Registry Network for Orphan Lung Disease (ARNOLD) experience

**DOI:** 10.1186/s13023-016-0389-z

**Published:** 2016-04-18

**Authors:** K. Casamento, A. Laverty, M. Wilsher, J. Twiss, E. Gabbay, I. Glaspole, A. Jaffe, On behalf the Pulmonary Interstitial Vascular Organisational Taskforce (PIVOT) of the Lung Foundation Australia

**Affiliations:** Sydney Children’s Hospitals Network, Sydney, Australia; Great Ormond Street Hospital for Children NHS Foundation Trust, London, England; Auckland District Health Board, Auckland, New Zealand; Starship Children’s Health, Grafton, Auckland New Zealand; Institute for Health Research, University of Notre Dame, Fremantle, WA Australia; Alfred Hospital, Melbourne, Australia; Monash University, Melbourne, Australia; School of Women’s and Children’s Health, UNSW Medicine, University of New South Wales, Sydney, NSW Australia; Department of Respiratory Medicine, Sydney Children’s Hospital Randwick, Sydney, NSW Australia

**Keywords:** Rare diseases, Lung disease, Registries, Prevalence, Australia, New Zealand

## Abstract

**Background:**

We investigated the feasibility of using an online registry to provide prevalence data for multiple orphan lung diseases in Australia and New Zealand.

**Methods:**

A web-based registry, The Australasian Registry Network of Orphan Lung Diseases (ARNOLD) was developed based on the existing British Paediatric Orphan Lung Disease Registry. All adult and paediatric respiratory physicians who were members of the Thoracic Society of Australia and New Zealand in Australia and New Zealand were sent regular emails between July 2009 and June 2014 requesting information on patients they had seen with any of 30 rare lung diseases. Prevalence rates were calculated using population statistics.

**Results:**

Emails were sent to 649 Australian respiratory physicians and 65 in New Zealand. 231 (32.4 %) physicians responded to emails a total of 1554 times (average 7.6 responses per physician). Prevalence rates of 30 rare lung diseases are reported.

**Conclusions:**

A multi-disease rare lung disease registry was implemented in the Australian and New Zealand health care settings that provided prevalence data on orphan lung diseases in this region but was limited by under reporting.

## Background

The Australian Therapeutic Goods Authority defines an orphan disease as “a rare disease, or condition, likely to affect not more than 2,000 individuals in Australia at any time” [1] , while in Europe it is defined as affecting less than 1 in 2000 people [2]. Despite individual diseases affecting only a small number of people, orphan diseases collectively contribute a significant portion of the overall burden of disease [3]. European studies estimate that one in three cases of severe disability in children is caused by a rare disease and combined, their prevalence is 6–10 % in the general population [4]. This would equate to approximately 1.2 million Australians, however definitive epidemiological data in the Australian population are limited [4, 5].

Orphan diseases may be varied in nature but they face similar barriers to their research and treatment development. Specifically, rare lung diseases are difficult to study because they are a heterogeneous group of diseases that often do not have a formal description in the International Classification of Diseases (ICD-10) [6, 7] , and in some cases, may be extremely rare. Defining cases for trials can be a challenge due to delayed diagnoses and poor clarity in diagnostic criteria [8]. Furthermore, poorly defined clinical endpoints can increase the cost of clinical trials [9]. Historically low cost-benefit values have been a major disincentive for pharmaceutical companies to invest in rare disease research [10], a factor that is now being mitigated by Orphan Drug legislation, developed in the US in 2002 [11], in Australia in 1998 [1] and the EU in 2000 [12]. More recently, initiatives such as the Creating Hope Act, passed as part of the Food and Drug Administrative Safety and Innovation Act in 2012 in the USA are aimed at enhancing drug discovery for people with a rare disease [13] by subsidising research and trials. In order to qualify for these schemes, disease specific prevalence data are essential to define qualifying diseases. Some pulmonary disease-specific registries have been successful internationally in providing these data; for example the Alpha One International Registry (AIR) [14] and Cystic Fibrosis Registries [15].

Despite being developed countries, Australia and New Zealand have unique characteristics in their population density distribution; their relatively small populations are dispersed over a large geographical area bringing further challenges to collaborative studies involving patients with rare and extremely rare diseases. In Australia, three National lung disease specific registries exist; the Australian CF Data Registry [16] and the relatively new Australian Idiopathic Pulmonary Fibrosis Registry [17] and Australian Bronchiectasis Registry.

Because of the urgent need for epidemiological data on rare lung diseases to support research funding and drug development in Australia and New Zealand we investigated the feasibility of using a single lung disease registry based on the web-based British Paediatric Orphan Lung Disease Registry (BPOLD) [18] to provide prevalence data for multiple orphan lung diseases in both the paediatric and adult population.

## Methods

This was a descriptive population-based study. A web-based registry entitled the Australasian Registry Network of Orphan Lung Diseases (ARNOLD) was established based on the model of BPOLD [18]. The ARNOLD website was created with Dreamweaver CS3 software (http://www.adobe.com) using HyperText Markup Language plus PHP scripting language (www.php.net). It was uploaded to a commercial server (www.siteground.com). All adult and paediatric respiratory physicians in Australia and New Zealand who were members of the Thoracic Society of Australia and New Zealand were invited to participate between July 2009 and June 2014. Initially quarterly emails were sent to Australian physicians until April 2011, after which emails were sent monthly. New Zealand physicians were invited to participate from 2011 with monthly emails. Members of the Pulmonary Interstitial Vascular Organisational Taskforce (PIVOT) of Lung Foundation Australia agreed on the inclusion of 30 rare lung diseases following face-to-face meetings and email correspondence ensuring a mixture of paediatric and adult diseases. Non cystic fibrosis bronchiectasis was not included as it was felt not to be an orphan disease in Australia and New Zealand.

Individual physicians were asked to respond via a simple link embedded within the email indicating whether they had or had not seen a patient (“nothing to report”) with one of the rare lung diseases in the previous month. If they answered in the affirmative they were automatically directed to the ARNOLD website and asked to enter the following details: reported disease (mandatory), patient initials, date of birth (DOB), postcode, medical record number and whether this was a “new case” or “follow-up” (all non-mandatory fields). Physician details, time and date were also logged automatically through the email link. Input rules such as mandatory fields and drop-down menus were developed to ensure high quality data. These details were transmitted securely using Secure Socket Layer (SSL), further encrypted using the MySQL ENCODE function (http://dev.mysql.com/doc/refman/5.1/en/encryption-functions.html#function_encode) and data were stored in a MySQL database located on the server. Statistical analyses were performed using Microsoft Excel and Microsoft Access software. Queries were used to first separate responses from Australian and New Zealand physicians and then paediatric and adult physicians. Duplicate data for the same patient were isolated by identifying records with a matching disease, patient initials, suburb and DOB and subsequently excluded from final calculations. Prevalence was calculated by dividing the total number of reports for each disease by the population estimates for 0–18 year-olds for paediatrics and older than 19 years old for adults for 2014 using data from the Australian Bureau of Statistics and Statistics New Zealand [19, 20]. This was then expressed as prevalence per million population over the 5 year study period.

### Ethics

This study was approved by the South Eastern Sydney Illawarra Area Heath Service Network Human Research Ethics committee for Australia (08/187) and the Multi-region Ethics Committee (MEC/11/03/026) for New Zealand.

## Results

Email invitations were sent to 649 Australian respiratory physicians and 65 in New Zealand. In response to approximately 32,300 emails, 231 (32.4 %) physicians responded to subsequent emails a total of 1554 times (4.8 % response rate). We received an average 6.73 responses per physician and an average of 33 responses per month. Thirty two participants (14 %) responded 13 or more times, 16 (7 %) responded 10–12 times, 24 (10 %) responded 7–9 times, 40 (17 %) responded 6–8 times and 118 (51 %) responded 1–3 times. Forty three paediatric physicians gave a total of 455 responses (average 10.6 responses per physician) and 188 adult physicians responded a total of 1099 times (average 5.8 responses per physician).

Participants responded with “nothing to report” a total of 1131 times (73 % of responses). In the positive responses there were five incidences of matching initials, postcode, DOB and disease and these were excluded from the final calculations as duplicates; however it is noted that 273 notifications (65 %) had missing patient identifiers. The status of ‘new diagnosis’ vs ‘follow up’ was reported on for 194 reports (46 %). Disease prevalence in Australia is listed in Table 1 and New Zealand in Table 2.

**Table 1:** Prevalence of rare lung diseases in Australia between 2009 and 2014– paediatric, adult and general population

Disease	Paediatric		Adult		Total population	
	No. of reports	Prevalence per million	No. of reports	Prevalence per million	No. of reports	Prevalence per million
Acute idiopathic eosinophilic pneumonia	1	0.18	2	0.12	3	0.13
Children’s interstitial lung disease (ChILD)	21	3.80	0	0.00	21	0.92
Chronic idiopathic eosinophilic pneumonia	0	0.00	7	0.40	7	0.31
Churg-Strauss vasculitis	0	0.00	9	0.52	9	0.39
Congenital Malformations of the Trachea	4	0.72	0	0.00	4	0.18
Cryptogenic organising pneumonia	0	0.00	21	1.21	21	0.92
Cystic lung lesions	12	2.17	5	0.29	17	0.74
Diaphragmatic Hernia	1	0.18	2	0.12	3	0.13
Diffuse panbronchiolitis	0	0.00	1	0.06	1	0.04
Drug reactions with eosinophilia	0	0.00	2	0.12	2	0.09
Extrinsic allergic alveolitis	0	0.00	16	0.93	16	0.70
Follicular bronchiolitis	1	0.18	0	0.00	1	0.04
Goodpasture syndrome	0	0.00	3	0.17	3	0.13
Hereditory haemorrhagic ataxia telangiectasia	0	0.00	7	0.40	7	0.31
Idiopathic pulmonary haemosiderosis	5	0.90	4	0.23	9	0.39
Langerhans cell histiocytosis	0	0.00	7	0.40	7	0.31
Lobar emphysema	7	1.27	1	0.06	8	0.35
Lymphangioleiomyomatosis	0	0.00	2	0.12	2	0.09
Obliterative bronchiolitis (not transplant related)	5	0.90	3	0.17	8	0.35
Other vasculitides incl. microscopic polyangiitis	1	0.18	7	0.40	8	0.35
Primary ciliary dyskinesia	4	0.72	2	0.12	6	0.26
Pulmonary alveolar proteinosis	3	0.54	5	0.29	8	0.35
Pulmonary amyloidosis	0	0.00	1	0.06	1	0.04
Tracheo-oesophageal fistula	4	0.72	1	0.06	5	0.22
Granulomatosis with polyangiitis	2	0.36	7	0.40	9	0.39
Totals	71	12.82	115	6.65	186	8.13

**Table 2:** Prevalence of rare lung diseases in New Zealand between 2009 and 2014– general population

Disease	No. of reports	Per million
Acute idiopathic eosinophilic pneumonia	0	0.00
Children’s interstitial lung disease (ChILD)	0	0.00
Chronic idiopathic eosinophilic pneumonia	0	0.00
Churg-Strauss vasculitis	1	0.22
Congenital Malformations - Trachea	0	0.00
Cryptogenic organising pneumonia	2	0.44
Cystic lung lesions	1	0.22
Diaphragmatic Hernia	0	0.00
Diffuse panbronchiolitis	2	0.44
Drug reactions with eosinophilia	0	0.00
Extrinsic allergic alveolitis	3	0.66
Follicular bronchiolitis	0	0.00
Goodpasture syndrome	0	0.00
Hereditory haemorrhagic ataxia telangiectasia	0	0.00
Idiopathic pulmonary hemosiderosis	1	0.22
Langerhans cell histiocytosis	0	0.00
Lobar emphysema	0	0.00
Lymphangioleiomyomatosis	0	0.00
Obliterative bronchiolitis (not transplant related)	0	0.00
Other vasculitides incl. microscopic polyangiitis	1	0.22
Primary ciliary dyskinesia	3	0.66
Pulmonary alveolar proteinosis	0	0.00
Pulmonary amyloidosis	0	0.00
Tracheo-oesophageal fistula	0	0.00
Granulomatosis with polyangiitis	0	0.00
Total	14	3.10

## Discussion

This study has demonstrated proof-of-concept that it is possible to use an online web based registry to aggregate numbers to create national prevalence data on 30 rare lung diseases in Australia and New Zealand. However physician engagement was low (32.4 %) indicating that the actual national prevalence rates are likely to be higher than reported.

Compared to single disease registries, those that collect data on multiple diseases have the benefit of cost-effectiveness, standardisation of data, and ease of use [21–23]. They capture information on many rare diseases that are potentially absent or misclassified in the ICD10 [7] and thus are not adequately registered by existing health systems. The Australian Federal and State Governments signed a National Health Information Agreement (NHIA) in 2013 [24] committing to better health information collection and dissemination. This registry therefore fills an important gap in collecting prevalence statistics in the Australian population and fulfils the objectives of the NHIA and represents the only prevalence data that exist for these diseases in Australia and New Zealand.

ARNOLD translated smoothly to the Australian and New Zealand healthcare settings from the already established UK BPOLD registry. In addition to being cost-effective, it is relatively simple to use with the functionality of a one-click secure reply. Input rules ensured that data entry was uniform and very little data maintenance was necessary. The EU Committee of Experts on Rare Diseases (EUCERD) recently convened to develop a set of core recommendations for rare disease registry design. They highlighted the need for easy exchange of data on an international level and a common standard of data collection [23], both of which are import features of the BPOLD/ARNOLD design.

The registry however faced a number of barriers. The data were limited by under-reporting of patient identifiers and other non-mandatory details. Only 35 % of notifications included details about postcode, DOB or patient initials, meaning that possible duplicate notifications could not be identified for the majority of the reports. This may have led to over-reporting of some rare conditions. Also, there were insufficient DOB data to distinguish paediatric and adult patients and so prevalence calculations were made by identifying the person reporting as either a paediatric or adult physician and we acknowledge that this may have limited the accuracy. The benefit of this information is that it gives an indication of the workload of paediatric physicians compared to adult physicians for these rare diseases.

The system has the capability of recording incidence data, however the ‘new diagnosis’ status was only recorded in 46 % of cases. This meant that the majority of cases could not be differentiated into new or existing diagnoses and incidence calculations could not be performed. Many of these limitations could be remedied by making more boxes mandatory at the data entry phase in the future, however it may compromise the system’s usability. A further limitation is the low response rate; of a possible 714 adult and paediatric physicians only 231 physicians (32.4 %) responded in contrast to the UK BPOLD where 64 % of physicians responded [18]. We speculate that one of the reasons the response rate of the UK BPOLD is higher than ARNOLD is that it involved only respiratory paediatrician respondents; paediatricians have been responding to monthly requests to report children with rare diseases since the establishment of the British Paediatric Surveillance Unit in 1986 [25] and as such, it is likely that they were more inclined to respond to requests to report monthly data compared to adult physicians. The Australian Paediatric Surveillance Unit [26] was established over 20 years ago and has a monthly response rate of approximately 95 % no such system exists for adult physicians.

Furthermore, it is possible that the list of invited physicians in ARNOLD contained many trainees who do not see rare lung disease patients in a clinic setting or that time pressures prevailed in a busy clinic setting thus creating a significant barrier to reporting patients to the registry.

A registry integrated with electronic medical records could bring efficiencies and would prevent overburdening busy clinicians, however the current systems depend on ICD-10 classifications from which many rare lung diseases are missing. This would also have ethical implications in gaining consent and the data would have to be completely de-identified before being collected by our database.

A final limitation was that specific disease definitions were not provided to reporting physicians and relied on individual physician diagnoses so there may have been some inconsistencies in reporting. Although this can be easily addressed for many rare lung diseases, for many rare lung diseases consensus opinion has not yet been reached on the diagnostic criteria and this is an enduring limitation in rare disease research.

The prevalence rates for each of the 30 rare lung diseases reported in this study are much lower than reported rates in other countries. For example, pulmonary alveolar proteinosis was found to have a prevalence of 0.35 per million in Australia, 3 per million in the BPOLD study [18] and between 4 and 40 per million internationally. The same pattern was seen consistently across many diseases. No cases were reported in New Zealand for 17 of 30 conditions. This could represent a genuinely lower rate of rare diseases in the Australasian population but it is more likely a reflection of low reporting rates from the comparatively low population of Australia and New Zealand.

In contrast, registries of single orphan lung diseases have had greater physician engagement. The Australian Idiopathic Pulmonary Fibrosis Registry has data on more than 600 patients since its establishment [17]. A key feature of its success is that each State and Territory in Australia has a research coordinator whose responsibility it is to aid physicians in entering the data onto the registry. Other successful examples of single orphan lung disease registries include cystic fibrosis [15] and, more recently children with interstitial lung disease (chILD) in the USA [27] and in the European Union (EU) [28]. A recent qualitative study that sought consensus opinions of 41 multidisciplinary rare disease experts highlighted need for physicians be convinced of the importance of rare disease registries in promoting and enhancing clinical research [29].

Despite these limitations, we have demonstrated that many physicians recognise the importance of contributing to a web-based rare disease registry which will help standardise care and allow for research. Even with low reporting, ARNOLD has provided data, for the first time, of the prevalence of patients with rare lung diseases in Australia and New Zealand. An important advantage of this multi-disease approach is that it is highly suitable for gathering data on extremely rare orphan diseases where there is no capacity to establish disease specific registries.

## Conclusions

We have demonstrated a proof of concept that it is feasible to obtain National data on the prevalence of multiple rare and extremely rare lung diseases using a web-based registry in Australia and New Zealand. These data will be important to pharmaceutical companies undertaking orphan drug development in Australasia and it will help define research priorities to benefit those many people living with a rare lung disease. The next step is to first evaluate the registry in order to understand how best to improve reporting and then adequately invest in this type of web-based registry approach to address the reporting barriers, increase awareness, aid data input to improve the accuracy of the data and make it sustainable.
